# A New Self-Healing Degradable Copolymer Based on Polylactide and Poly(p-dioxanone)

**DOI:** 10.3390/molecules28104021

**Published:** 2023-05-11

**Authors:** Laifa Tong, Mi Zhou, Yulong Chen, Kai Lu, Zhaohua Zhang, Yuesong Mu, Zejian He

**Affiliations:** Material Science and Engineering, College of Materials Science and Engineering, Zhejiang University of Technology, Hangzhou 310000, China; 13586263187@163.com (L.T.); zhoumi@zjut.edu.cn (M.Z.); lk_1092647817@163.com (K.L.); zhaohua119@126.com (Z.Z.); 2112025110@zjut.edu.cn (Y.M.); hezejian@zjut.edu.cn (Z.H.)

**Keywords:** Diels–Alder reaction, poly(p-dioxanone), polylactide, crystallization behavior, self-healing

## Abstract

In this paper, the copolymerization of poly (p-dioxanone) (PPDO) and polylactide (PLA) was carried out via a Diels–Alder reaction to obtain a new biodegradable copolymer with self-healing abilities. By altering the molecular weights of PPDO and PLA precursors, a series of copolymers (DA2300, DA3200, DA4700 and DA5500) with various chain segment lengths were created. After verifying the structure and molecular weight by 1H NMR, FT-IR and GPC, the crystallization behavior, self-healing properties and degradation properties of the copolymers were evaluated by DSC, POM, XRD, rheological measurements and enzymatic degradation. The results show that copolymerization based on the DA reaction effectively avoids the phase separation of PPDO and PLA. Among the products, DA4700 showed a better crystallization performance than PLA, and the half-crystallization time was 2.8 min. Compared to PPDO, the heat resistance of the DA copolymers was improved and the Tm increased from 93 °C to 103 °C. Significantly, the rheological data also confirmed that the copolymer was self-healing and showed obvious self-repairing properties after simple tempering. In addition, an enzyme degradation experiment showed that the DA copolymer can be degraded by a certain amount, with the degradation rate lying between those of PPDO and PLA.

## 1. Introduction

In recent years, the development of degradable polymer materials has played an increasingly important role in improving people’s lives, as well as reducing environmental pollution and constructing implantable materials [1,2,3]. For instance, polylactide (PLA) is frequently used as a surgical suture and for stent implantations because of its excellent biocompatibility, biodegradability and processing properties [4,5,6], while poly(p-dioxanone) (PPDO) is often used as a surgical suture and stent implantation owing to its stronger biodegradability and biocompatibility [7,8,9,10]. However, different application scenarios have led to higher requirements for polymer properties, making it more challenging for single degradable polymer materials to satisfy human needs [11,12]. For instance, PPDO has good crystallinity because of its good molecular chain flexibility [13,14], but PLA has drawbacks such as a poor toughness and a sluggish crystallization rate [15,16,17]. However, there is little compatibility between them, so direct blending would not produce the desired material. Therefore, a convenient way to improve the overall performance of degradable polymers is through the straightforward copolymerization of two polymers [18,19,20].

The following examples [21] are where copolymerization’s impact on biomedicine is most clearly visible. First, the copolymerization of PPDO and PLA can lead to control of the copolymer’s degradation cycle, which has a significant impact on in vivo degradation. Second, the ratio of monomers may be regulated to improve PPDOs comprehensive performance, which can be used in a variety of application scenarios. Finally, researchers can prepare corresponding copolymer materials in accordance with various application requirements because materials produced by copolymerization are more frequently used than homopolymers.

The Diels–Alder reaction is a [4 + 2] cycloaddition reaction between dienes and dieno-philes [22,23]. DA adducts are formed at 50~90 °C, and the inverse Diels–Alder reaction (r-DA reaction) occurs at 100–130 °C. When cooling to a low temperature, the broken DA bond re-undergoes the DA reaction and forms DA adducts [24,25,26,27]. Due to its thermal reversibility and mild reverse reaction conditions, the DA reaction is regarded as a practical method to construct self-healing materials. Fred et al. [28] used the DA reaction to form a transparent polymer material. The material is a tough solid near room temperature and has mechanical properties comparable to those of commercial epoxy resins. At temperatures above 120 °C, the crosslinking point is disconnected and can be re-connected after cooling. In 2016, Xia et al. [29] also prepared a re-modellable, recyclable and self-repairing polysiloxane elastomer by cross-linking maleimide-functionalized polysiloxane with furan-end-functionalized polysiloxane. Therefore, it seems feasible to construct self-healing polymeric materials based on PPDO and PLA with the aid of the efficient DA reaction.

In this study, a series of DA copolymers were synthesized by the DA reaction of PPDO prepolymer with PLA prepolymer. The molecular weight of the experimental PPDO prepolymer and the PLA prepolymer was about 1:1. DA copolymers (DA2300, DA3200, DA4700 and DA5500) were obtained by altering the molecular weight of prepolymers. The structure of the product was characterized by ^1^H NMR, FT-IR and GPC. In addition, the thermal stability, crystallization ability, self-healing properties and degradation properties of DA products were studied by DSC, XRD, POM, rheological measurements and enzymatic degradation.

## 2. Results and Discussion

### 2.1. Synthesis and Characterizations

**Scheme 1:**
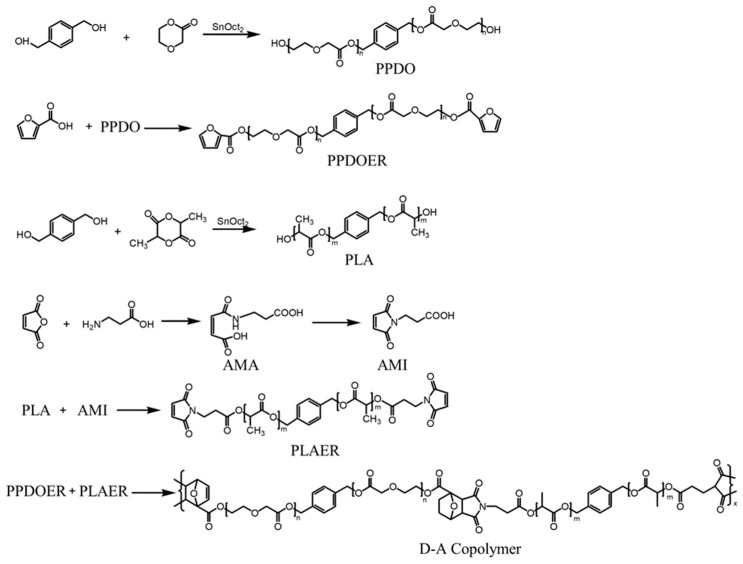
Synthetic routes towards DA copolymers.

**Figure 1:**
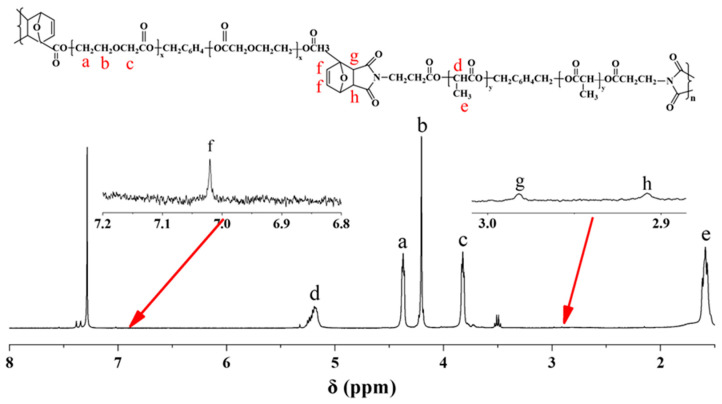
^1^H NMR spectrum of the DA copolymer.

The synthesis routes to DA copolymers are shown in Scheme 1 and the ^1^H NMR spectra of all intermediate products are shown in Figure S1. In Figure S1a, PPDO2300 is used as an example, and the ratio of integrated peak areas of a, b and c is 1:1:1, which corresponds exactly to the structure of PPDO. Meanwhile, the ratio of integrated peak areas of a and d showed that the molecular weights of the four PPDO products were 2300, 3200, 4700 and 5500, respectively, which was also confirmed by the ratio of integrated peak areas of a, b (furan rings) and e in Figure S1b. On the other hand, the maleimide group grafting process of PLA mainly includes the synthesis of PLA and AMI and the esterification reaction between them. Figure S1c,d demonstrates the structures of PLA and AMI, and the molecular weights of PLA products were also calculated as 2300, 3200, 4700 and 5500, respectively, by the ratio of integrated peak areas of b and c in Figure S1c. Subsequently, the presence of peaks a, b and c in Figure S1e also proved the successful grafting of maleimide groups and the successful synthesis of PLAER. The ^1^H NMR spectrum of the DA copolymer is shown in Figure 1, the peaks of a-c come from PPDO, and d-f correspond to the peaks of PLA and furan ring, in addition, the presence of peaks g and h indicates the successful completion of the DA reaction and the linkage between PLA and PPDO by DA bonds.

Each synthesis step could also be confirmed by the FT-IR spectrum (Figure S2). In Figure S2a, PPDOER showed a relatively small peak at ~765 cm^−1^, which is the characteristic peak of furan groups grafted to PPDO after esterification. The new peak at 1709 cm^−1^ of PLAER in Figure S2b was attributed to the two carbonyl groups of the maleimide group. More importantly, in Figure S2c, the presence of a DA bond made a new peak appear in the spectrum of the copolymers at 1748 cm^−1^, indicating the successful synthesis of DA copolymers. The GPC spectrum of DA copolymers is shown in Figure S3, and it can be found that the molecular weight of the copolymer increased with the increase in the molecular weight of the PPDO and PLA macromolecular monomers. Although the molecular weight distribution was wide, only a single peak can be observed in the spectra, proving that the composition of the copolymers was as expected.

### 2.2. Thermal Stability of DA Copolymer

**Figure 2:**
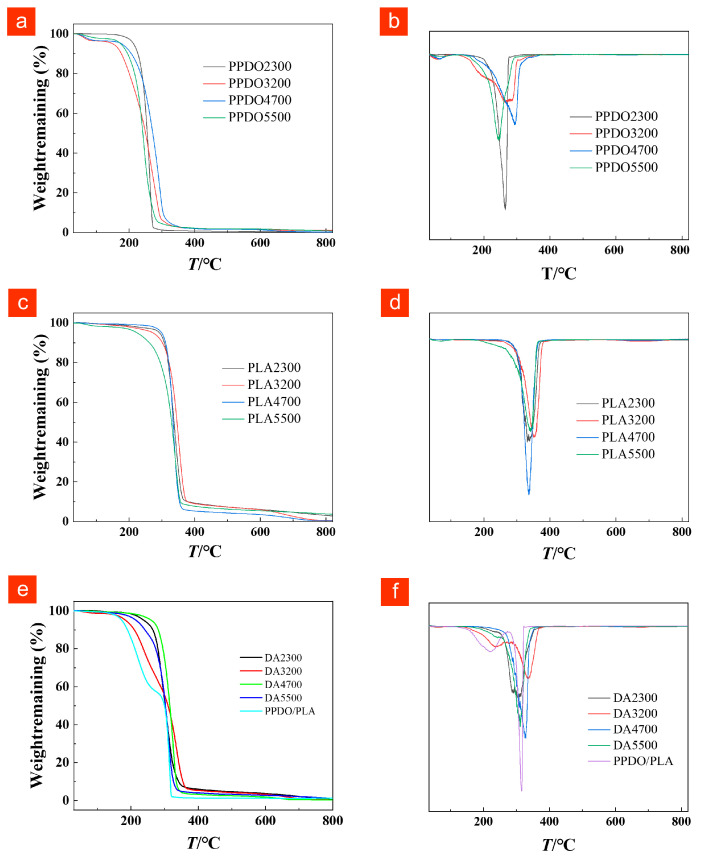
TG (**a**,**c**,**e**) and DTG **(b**,**d**,**f**) spectra of each sample: (**a**,**b**) PPDO; (**c**,**d**) PLA; (**e**,**f**) DA.

The thermal stabilities of PPDO, PLA and DA copolymers were measured by TGA in a nitrogen atmosphere, and the relevant information is summarized in Figure 2 and Table S1. The thermal degradation process of PPDO was generally divided into three stages. In the first stage, within the range of 30–200 °C, the weight loss was mainly due to the evaporation of adsorptive water and crystalline water of the samples because of physical dehydration, with a mass loss of about 7 %. The second stage consists of weight loss in the range of 200–400 °C, which was mainly attributed to the thermal degradation fracture of molecular chains. In the third stage, carbonization occurred at about 500 °C, and the final residue rate of all samples was about 6 % at 500 °C. Compared with PPDO, the initial decomposition temperature (*T*_5%_) of DA copolymers increased from 150 °C to 260 °C due to the introduction of a PLA segment. Meanwhile, the maximum thermal decomposition temperature (*T*_max_) increased from about 275 °C to 325 °C, and with the increase in molecular weight, *T*_max_ tended to move towards a higher temperature. In addition, compared to the two peaks of the PPDO/PLA blend in the DTG spectrum, DA copolymers only have one peak, except for DA3200, demonstrating the successful preparation of DA copolymers again. As for DA3200, the occurrence of a r-DA reaction may be responsible for the two peaks. In general, compared to PPDO, DA copolymers show higher thermal stabilities, with the thermal stability gradually increasing with increasing molecular weight.

### 2.3. Crystallinity of DA Copolymers

**Figure 3:**
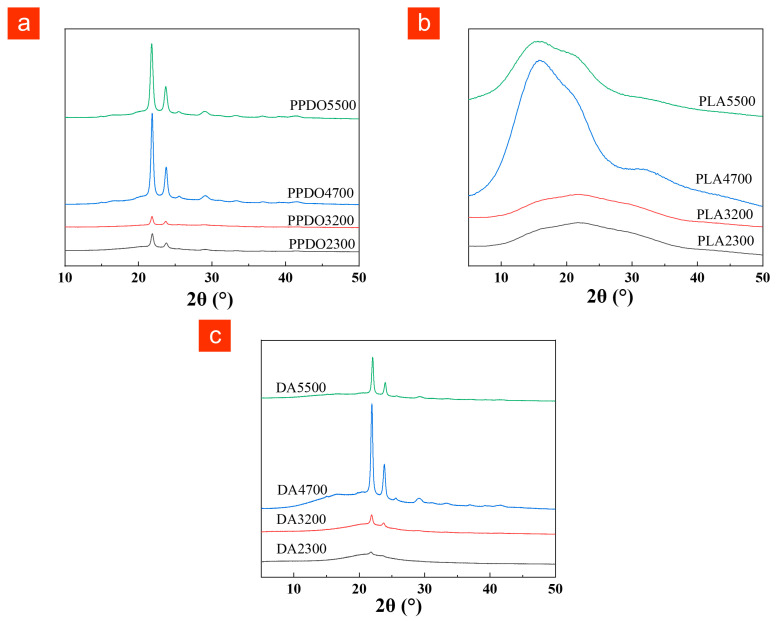
XRD spectra of each sample: (**a**) PPDO; (**b**) PLA; (**c**) DA product.

Due to both PLA and PPDO being crystalline, the crystallinity of the DA copolymer is also of great interest. Firstly, XRD was used to characterize the crystal structure of the polymers. In Figure 3, it can be observed that PPDO has strong diffraction peaks at the diffraction angles of 21.9°, 23.8° and 29.1°, and the corresponding d-spacing is calculated as 4.05 (d210), 3.74 (d020) and 3.06 (d310), respectively, while PLA samples only have a large and wide peak between 10° and 30°, indicating their polycrystalline structure and relatively poor crystallization ability. Meanwhile, the crystallization ability of PLA seems to be related to its molecular weight, and a strong diffraction peak at 15.7° could be observed when the molecular weight increased to about 5 kDa. As for the DA copolymer, its XRD spectrum (Figure 3c) appeared to be a superposition of those of both PPDO and PLA. However, among the two, PPDO exhibits much stronger crystal diffraction peaks than PLA due to its stronger crystallization ability. In addition, the effect of molecular weight was also obvious, i.e., the diffraction peak of PPDO in the copolymers was first enhanced and then weakened, which may be attributed to the fact that the addition of PLA affects the regular arrangement of molecular chains, making the overall crystallization ability of the polymer decrease.

**Figure 4:**
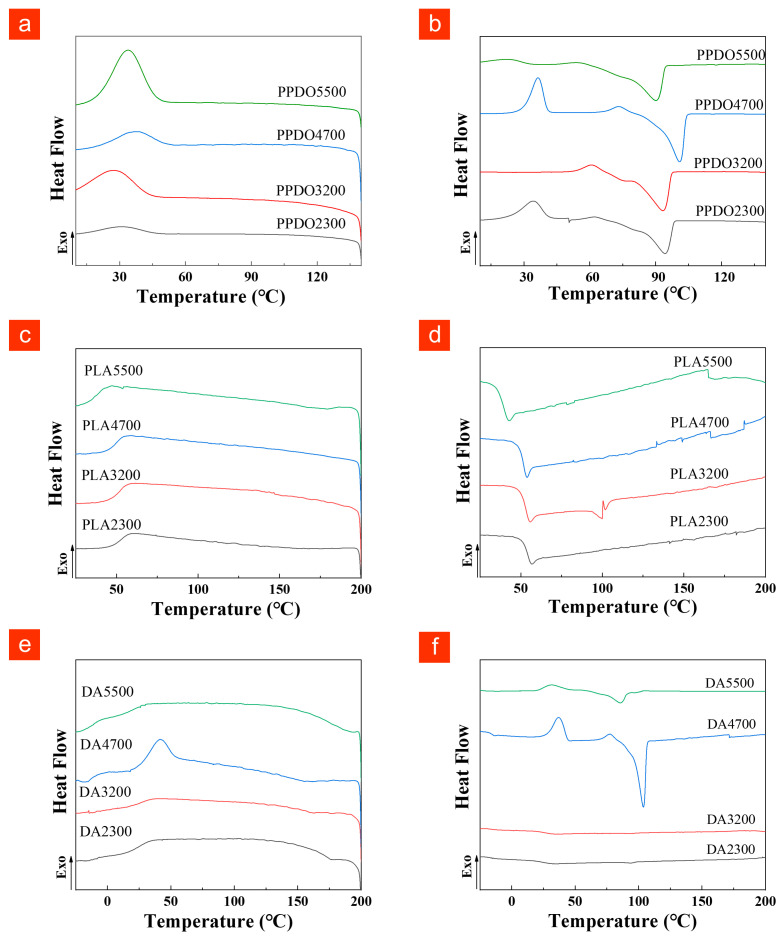
DSC curves of each sample: (**a**) cooling and (**b**) heating scans of PPDO; (**c**) cooling and (**d**) heating scans of PLA; (**e**) cooling and (**f**) heating scans of DA copolymers.

Meanwhile, DSC was also used to characterize the thermal and crystallization properties of the copolymers. The differential scanning calorimetry curves of all samples are shown in Figure 4 and relevant data are listed in Table 1. As shown in Figure 4a,b, crystallization exothermic peaks appeared in all PPDO samples, and exothermic crystallization peaks appeared at 34.17 °C and 36.46 °C, respectively, for PPDO2300 and PPDO4700 in the subsequent heating curves. This phenomenon may be due to incomplete crystallization during the previous cooling scan and the occurrence of cold crystallization during heating. In general, the melting point of PPDO increases from 93 °C to 100 °C with increasing molecular weight. As for PPDO5500, more PDO monomers may be the main cause of the increase in lattice defects and the decrease in thermal stability. However, under the same conditions, PLA has no obvious crystal peak in the spectrum (Figure 4c,d) due to its weak crystallinity. As for the DA copolymer, only DA4700 and DA5500 showed obvious crystallization exothermic peaks, while the other samples did not (Figure 4e,f). This may be due to the fact that the smaller macromonomer causes increased mixing of PPDO and PLA after polymerization, making PLA have a greater impact on the crystallization ability of PPDO. In the case of DA4700 and DA5500, they also failed to crystallize completely during the cooling process, and there was also a cold crystallization peak in the heating curve. According to Table 1, with the increasing molecular weight of the homopolymer, the crystallinity (*X*c) of the copolymer increased to about 20% compared to that of PLA homopolymer. Compared to the PPDO homopolymer, the melting point of the copolymer also improved. The results show that the increase in molecular weight of PPDO improves the crystallization properties of the DA copolymer and the introduction of PLA chain segments improves the thermal stability of PPDO, which is consistent with the results of XRD and TG above.

Detailed crystallization peak data, such as crystallization temperature (*T*_c_), crystallization enthalpy (Δ*H*_c_), recrystallization temperature (*T*_cc_), recrystallization enthalpy (Δ*H*_cc_), melting point (*T*_m_), enthalpy of melting (Δ*H*_m_) and crystallinity (*X*_c_), obtained from all DSC experiments are listed in Table 1. The calculation formula is shown in (1):

(1) $$X_{c}=\frac{\Delta H_{f}}{\Delta H_{f}^{{}^{\circ}}\times A\left(\omega t\%\right)}*100\%$$

where Δ*H_f_* and Δ*H_f_*^°^ are the melting heat of crystallization of component A in the sample and the melting enthalpy of crystallization of 100% crystalline A homopolymer (the melting enthalpy of fully crystallized PLA is reported to be 93 J/g and that of PPDO is 141 J/g), respectively, and *A* (*wt*%) represents the mass percentage of component A in the sample. Here, the mass ratio of PLA to PPDO is 1:1, so the melting enthalpy of complete crystallinity of copolymer is calculated as 117 J/g on average.

**Figure 5:**
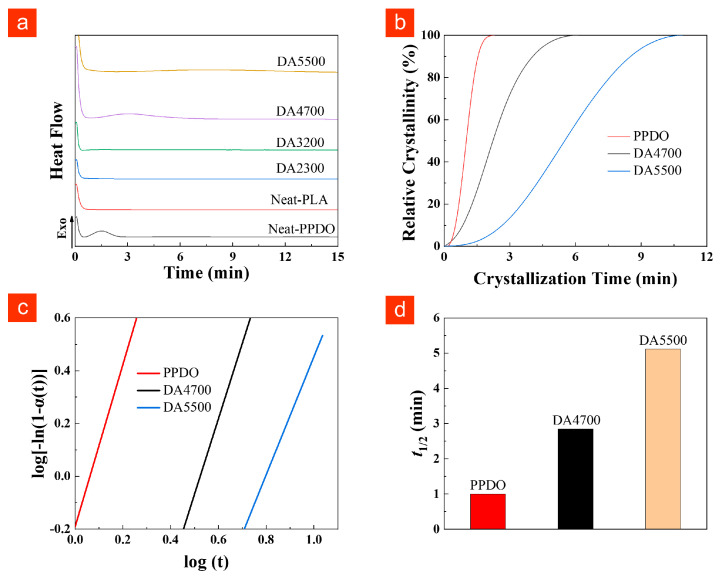
(**a**) DSC heat flow as a function of time; (**b**) the relative crystallinity as a function of time; Avrami curve equation (**c**) and half−crystallization time (*t*_1/2_) (**d**) of each component.

Moreover, the isothermal crystallization exothermic curves of each sample are also shown in Figure 5a, and it can be seen that only PPDO, DA4700 and DA5500 have crystallization peaks. Among the three, the crystallization rate of PPDO is the fastest, followed by DA4700, and DA5500 is the slowest (Figure 5b), which may be due to the fact that macromolecules with a larger molecular weight need a longer time to arrange themselves into regular chains and form crystals. According to the Avrami equation, the half crystallization time (*t*_1/2_) of PPDO is 0.99 min, while those of DA4700 and DA5500 are 2.85 min and 5.11 min, respectively (Figure 5c,d and Table S2).

**Figure 6:**
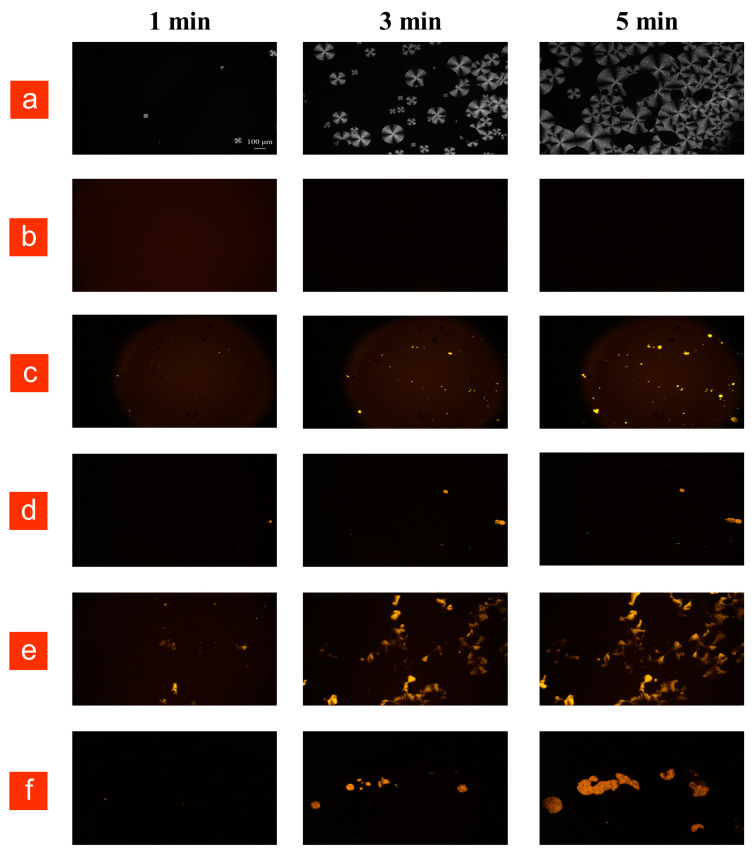
Time evolution of POM micrographs of crystal morphologies for each sample: (**a**) PPDO in 30 °C; (**b**) PLA in 80 °C; (**c**) DA2300 in 50 °C; (**d**) DA3200 in 50 °C; (**e**) DA4700 in 50 °C; (**f**) DA5500 in 50 °C.

The time evolution of POM micrographs of the polymers is shown in Figure 6. For PPDO, a spherocrystal with a clear Maltese extinction cross and alternating light and dark concentric rings, which is consistent with the literature, can be observed by isothermal crystallization at 30 °C (Figure 6a). Meanwhile, PLA did not show any crystal structure after isothermal crystallization at 80 °C for 15 min, which was consistent with the results of XRD and DSC. As for the DA copolymer, only DA4700 could be observed to have a spherulite structure with a weak Maltese cross and light and dark concentric rings (Figure 6e), while a few crystals can be seen in others. In particular, DA2300 and DA3200, with small molecular weights, took a longer time to complete the crystallization, which was consistent with the results of DSC. In summary, with the increase in molecular weight, the crystallinity of the DA copolymer generally showed a trend of enhancement, and DA4700 showed the best crystallinity, which was mainly dependent on PPDO segments.

### 2.4. Rheological Properties and Self-Healing Properties

The relationship between apparent viscosity (η) and shear rate was obtained by determining the shear rate of DA copolymers with different macromonomer molecular weights, as shown in Figure S4. The apparent viscosity decreased with the increase in shear rate, and shear thinning behavior can be observed clearly, indicating that the copolymer is a non-Newtonian pseudoplastic fluid. Moreover, η clearly decreased with the increase in molecular weight of the macromonomers at shear rates of 0.1–1000 s^−1^, which may be attributed to the fact that the longer flexible PPDO segment weakens the entanglement between molecular chains, making the copolymer molecules more susceptible to untangle and slip under shear stress. In general, with the increase in molecular weight of PPDO and PLA, the influence of PPDO on viscosity is dominant.

**Figure 7:**
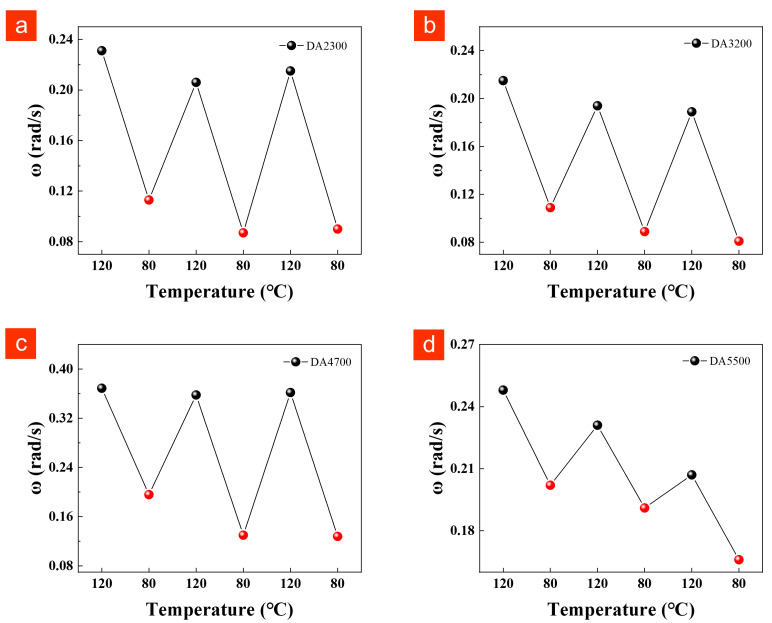
The intersection of G′ and G″ of DA copolymers as a function of temperature: (**a**) DA2300; (**b**) DA3200; (**c**) DA4700; (**d**) DA5500.

In addition, the DA copolymer was synthesized by linking PPDO and PLA chain segments with a DA bond, which is a dynamic covalent bond and reversibly broken at 120 °C. Thus, the self-healing property of the DA copolymer is predicted. The four groups of final DA products were scanned three times at different temperature cycle angular frequencies. The four groups of DA copolymer were cyclically scanned three times at different temperatures (80 and 120 °C) to obtain the curves of storage modulus (G′) and loss modulus (G″) with respect to angular frequency (Figures S5 and S6). When G′ is greater than G″, the polymer mainly behaves as a solid, while, vice versa, it behaves as a liquid, which can also be considered here as the breaking of the molecular chains under shear. The intersections of the three cycles in the low frequency region at 120 °C and 80 °C were recorded, respectively, to plot against temperature, and Figure 7 was obtained. It can be found that after completing a cycle, the intersection at 80 °C can basically return to the same level as before, indicating that the previously damaged DA bond can be recovered at high temperatures, and thus the copolymer has a self-healing ability. Since there are more DA bonds in the copolymer with small molecular weights (such as DA2300), the angular frequency of the intersection decreased less after cycling, which also indicates that the self-healing property of the copolymer comes from the DA bonds and can be strengthened by the regulation of the molecular structure.

### 2.5. Degradation Properties

**Figure 8:**
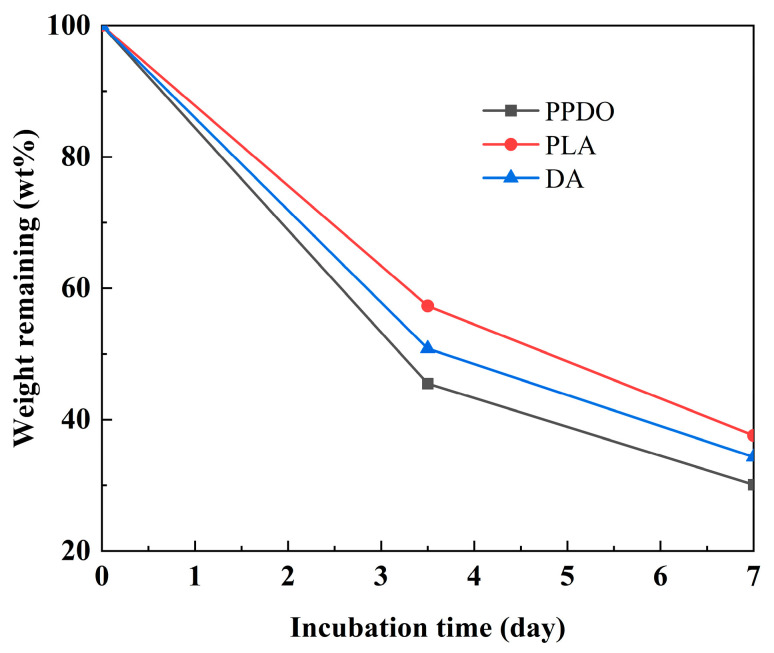
The change in residual mass with time during degradation of each sample.

The degradation performance of polymer materials is an important index for medical applications. The enzymatic degradation of the PPDO, PLA homopolymer and DA copolymer by protease K was investigated, as shown in Figure 8. During these experiments, a high level of protease K activity was obtained by refreshing the enzyme solution and applying higher temperatures. The results showed that the degradation rate of PPDO was the highest and that of PLA was the slowest, while the degradation rate of the DA copolymer was between those of the homopolymers PPDO and PLA. These results were mainly because the hydrophilicity of PPDO is better, so its degradation is faster. After 7 days of degradation testing, the weight retention rate of the DA material was 34.3%, lower than that of PLA (37.6%) and higher than that of PPDO (30.1%). The block copolymerization of PPDO and PLA was achieved through DA bonds. The new material inherits some of the physicochemical properties of different blocks. The biodegradability of the material has been well preserved.

## 3. Materials and Methods

### 3.1. Materials

Lactide (LA, 98%) and p-dioxanone (PDO, 98%) were purchased from Beijing Enokai Technology., Beijing, China and Shanghai MacLean Biochemical Technology., Shanghai, China, respectively. Stannous 2-ethyl hexanoate (Sn(Oct)_2_) and furfuric acid were obtained from Shanghai Aladdin Biochemical Technology Co., LTD., Shanghai, China. Proteinase K was obtained from MedChemExpress., Shanghai, China. Maleic anhydride and β-alanine were obtained from Adama Reagent Co., LTD., Shanghai, China. Other chemicals were of analytical grade and used without further purification.

### 3.2. Synthesis of PPDO Modified by Furan Groups

The monomer PDO (3.0 g, 29.4 mmol) and the initiator 1,4-benzenedimethanol were placed into a round-bottomed flask in a certain proportion, and stannous caprylate was also added as the catalyst [30]. The experimental ratios and conditions are shown in Table S3. After the reaction finished, trichloromethane was added to dissolve the solid in flask, and the solution was precipitated in excess methanol. After filtration, the precipitate was washed with ether and dried under vacuum overnight to obtain white powdery products (PPDO2300, PPDO3200, PPDO4700 and PPDO5500). ^1^H NMR (400 MHz, CDCl3, δ): 7.39 (s, Ar-H), 4.38 (t, -CH2-OOC-), 4.21 (s, -CH2-COO-), 3.82 (t, -CH2-O-).

Subsequently, in a dry box under an Ar atmosphere, to a dry flask containing a certain amount of furoic acid, PPDO, EDC and DMAP was added a suitable amount of ethanol [31,32]. The specific experimental ratios and conditions are shown in Table S4. After the reaction, the products were precipitated with ether and dried in a vacuum oven at 40 °C to obtain the corresponding products (PPDO2300ER, PPDO3200ER, PPDO4700ER and PPDO5500ER). ^1^H NMR (400 MHz, CDCl3, δ): 7.61 (s, =CH-O-), 7.39 (s, Ar-H), 6.79 (s, =CH-), 4.38 (t, -CH2-OOC-), 4.21 (s, -CH2-COO-), 3.82 (t, -CH2-O-).

### 3.3. Synthesis of PLA Modified by Maleimide Groups

The monomer LA (5.0 g, 34.7 mmol) and the initiator 1,4-benzenedimethanol were placed into a round-bottomed flask in a certain proportion, and stannous caprylate was also added as the catalyst [33,34]. The specific experimental ratios and conditions are shown in Table S5. During the reaction, the products (PLA2300, PLA3200, PLA4700 and PLA5500) were obtained by precipitation with excess methanol, washing with ether and drying under vacuum overnight. ^1^H NMR (400 MHz, CDCl3, δ): 7.35 (s, Ar-H), 5.18 (m, >CH-CO-), 1.53 (s, C-CH3).

In a 250 mL flask, β-alanine (8.01 g, 90 mmol) and maleic anhydride (10.58 g, 0.11 mol) were mixed in glacial acetic acid (108 mL) and stirred for 7 h. The crude AMA was obtained by evaporation of solvent, and pure AMA was obtained by recrystallization with methanol. Then, AMA was suspended in toluene (150 mL), and the suspension was stirred at 130 °C for 3 h. After cooling to room temperature, a yellow precipitate was obtained by evaporation. The crude product was acidized by HCl, extracted by ethyl acetate and recrystallized by ethyl acetate to obtain pure maleimide propionic acid (AMI) [35].

Subsequently, in an Ar atmosphere, to a dry flask containing a certain amount of AMI, PLA, EDC and DMAP was added a suitable amount of dichloromethane. The reaction ratios and conditions are shown in Table S6. After 24 h of reaction at room temperature, a solid was precipitated with ether and dried in vacuum oven at 40 °C to obtain the corresponding products (PLA2300ER, PLA3200ER, PLA4700ER and PLA5500ER). ^1^H NMR (400 MHz, C3D6O, δ): 6.99 (s, =CH-CO-), 5.18 (m, >CH-CO-), 3.16 (s, >N-CH2-), 2.92 (s, -CH2-COO-), 1.53 (m, C-CH3).

### 3.4. Synthesis of DA Copolymer

The PPDOER and PLAER products prepared above were mixed in dichloromethane at the same molecular weight. The reaction ratios and conditions are shown in Table S7. By controlling the maleimide/furan molar ratio at about 1.0, the DA reaction was completed at 60 ℃ to obtain four groups of DA products (DA2300, DA3200, DA4700 and DA5500) [36,37,38]. ^1^H NMR (400 MHz, CDCl_3_, δ): 7.03 (s, =CH-C), 5.18 (m, >CH-CO-), 4.38 (t, -CH_2_-OOC-), 4.21 (s, -CH_2_-COO-), 3.82 (t, -CH_2_-O-), 2.98 (s, -CH<), 2.91 (s, -CH<), 1.53 (m, C-CH_3_).

### 3.5. Characterization

#### 3.5.1. Proton Nuclear Magnetic Resonance Spectroscopy (^1^H NMR)

A Bruker Avance-400 NMR spectrometer (400 MHz) was used to obtain ^1^H NMR spectra of the samples. The solvent of the sample was deuterated chloroform (CDCl_3_) or deuterated acetone (C_3_D_6_O) as required, and tetramethylsilane was used as the internal standard.

#### 3.5.2. Fourier Transform Infrared Spectroscopy (FT-IR)

A Nicolet 6700 Fourier transform infrared spectrometer was used to measure the infrared spectra of the samples. The test method was as follows: the sample was ground into powder after drying in a vacuum oven, and the attenuated total reflection method (ATR) was used. The measurement range was 4000–650 cm^−1^.

#### 3.5.3. Gel Permeation Chromatography (GPC)

The molecular weights and polydispersity of the DA copolymer were determined using a THF GPC setup operating at 35 °C and comprising Styragel HR1, HR2 and HR4 columns, a Waters 2414 refractive index detector and a Waters 1515 pump. The GPC eluent was THF (2 *v*/*v*% triethylamine) at a flow rate of 1.0 mL/min.

#### 3.5.4. X-ray Diffraction

The changes in the crystallization properties of PPDO and PLA before and after the copolymerization were observed via a Japanese Ultima IV series composite X-ray diffractometer. The samples were dried at 25 °C, and X-ray diffraction patterns were obtained under Cuka radiation generated at 40 kV and 40 Ma. The scanning speed was 3°/min and the diffraction angle range (2θ) was 3–50°.

#### 3.5.5. Thermogravimetric Analysis (TG)

The thermal stability of the target polymer was determined via a Q5000IR thermal analyzer (TA Company of America). Under the protection of a nitrogen flow of 20 mL/min, the samples were raised from room temperature to 820 °C at a heating rate of 20 °C /min, and the data were recorded by a computer.

#### 3.5.6. Differential Scanning Calorimetry (DSC)

An L-700 differential scanning calorimeter (Toledo, Mettler, Switzerland) was used to determine the non-isothermal crystallization behavior of each polymer.

Non-isothermal crystallization test conditions: Under a nitrogen atmosphere, the gas flow rate was 50 mL/min, the temperature was increased from room temperature to a specific temperature (PPDO series products: 140 °C and PLA and DA series products: 200 °C) at a rate of 10 °C/min and the thermal history was eliminated by 5 min of heat retention. Then, the temperature was lowered to −30 °C at a cooling rate of 10 °C/min. Then, the temperature was raised to a specific temperature at a heating rate of 10 °C/min, and the non-isothermal DSC curves of the samples were recorded.

Isothermal crystallization test conditions: Under a nitrogen atmosphere, the gas flow rate was 50 mL/min and the temperature rose from room temperature to a specific temperature (PPDO: 140 °C and PLA and DA: 200 °C) at a rate of 10 °C /min for 5 min to eliminate the thermal history. The temperature was then rapidly reduced to crystallization temperature (30 °C for PPDO, 90 °C for PLA and 50 °C for DA) at a cooling rate of 50 °C/min and held for 15 min. The samples were then cooled to room temperature at a cooling rate of 10 °C/min, and then raised to a specific temperature at 10 °C/min. The isothermal DSC curves of the samples were recorded.

#### 3.5.7. Polarizing Optical Microscopy (POM)

The spherulite morphology of isothermal crystals was investigated by POM with a hot table. A small amount of the samples was placed on the hot table of the polarizing microscope, and the temperature was rapidly raised to 150 °C. The samples were kept at this temperature for 3 min to completely melt. Then, the samples were pressed into films and rapidly cooled to the desired crystallization temperature to observe the crystallization.

#### 3.5.8. Rheological Property

The rheological behavior of the composites was characterized by a stress-controlled rotational rheometer (MCR 302), and shear rate scanning and angular frequency scanning were performed. A parallel plate clamp with a diameter of 25 mm and a spacing of 1 mm was adopted.

Shear rate scanning conditions: the temperature was set at 110 °C, the strain was fixed at 1%. Additionally, the shear rate was scanned within 0.1–1000 s^−1^ and the viscosity was measured.

Angular frequency sweep conditions: the strain was fixed at 1% and the angular frequency scanning range was 0.01–628 rad/s. The energy storage modulus (G′) and loss modulus (G″) were measured at 120 °C and 80 °C, respectively. Three heating–cooling cycles were measured and the data were recorded.

#### 3.5.9. Degradation Property

Enzymatic degradation of each sample was carried out using proteinase K. In each trial, 50 mg of the sample was dispersed in 5 mL of 0.1 M Tris/HCL buffer (pH 8.5) containing protease K (2 mg/mL) and incubated at 50 °C. After 3.5 days incubation, the solution was filtered, the residue captured on the filter was washed with plenty of distilled water, then lyophilized, weighed, dispersed again in 5 mL fresh enzyme solution and incubated again at 50 °C for 3.5 days. The remaining solid mass was weighed [39,40].

## 4. Conclusions

In this paper, a series of biodegradable copolymers with self-healing abilities were synthesized by the DA reaction of PPDO and PLA prepolymers, and the chain segment length of the copolymers was regulated by the molecular weight of prepolymers (2300, 3200, 4700 and 5500). The performance of the DA copolymer was compared to that of PPDO and PLA homopolymers, and the former exhibited an improved thermal stability and heat resistance compared to the latter. For instance, copolymerization increased the Tm of the DA copolymer from 93 °C (pure PPDO) to 103 °C. Furthermore, due to the introduction of PPDO, the crystallization ability of the copolymer was greatly improved compared to PLA, with the crystal structure mainly depending on the PPDO segments. Among the copolymers of varying molecular weights, DA4700 exhibited the best crystallization ability with a *t*_1/2_ of approximately 2.8 min. Significantly, a rheological analysis revealed the copolymers’ self-healing properties. In addition, an enzyme degradation experiment showed that the DA copolymer was degraded by a certain amount, and the degradation rate was between that of PPDO and PLA. It was found that the segment composition of PPDO and PLA may change the thermal and crystalline properties of the copolymer. In addition, the crystallization properties of the copolymer can further affect the mechanical properties of medical materials such as hemostatic clips and surgical sutures, which provides a route for expanding the applications of PPDO. In conclusion, PPDO will be a focus of degradable polymer research for a considerable amount of time as a novel degradable polyether ester. In the future, the range of materials based on PPDO will become more and more extensive, and it may be possible to develop new degradable composite materials based on PPDO to replace most plastics and reduce environmental pollution.

## Figures and Tables

**Table 1:** Non-isothermal crystallization parameters of PPDO and DA4700.

Samples	*T*_c_ (°C)	Δ*H*_c_ (J/g)	*T*_cc_ (°C)	Δ*H*_cc_ (J/g)	*T*_m_ (°C)	Δ*H*_m_ (J/g)	*X*_c_
PPDO2300	30.54	10.54	34.17	32.56	93.15	76.06	30.6
PPDO3200	27.59	28.85	60.55	7.73	94.08	70.88	26.1
PPDO4700	37.92	23.72	36.46	40.24	100.70	68.21	45.4
PPDO5500	33.84	60.23	-	-	89.90	61.37	42.7
DA4700	37.06	10.02	37.06	15.04	103.95	32.85	21.4
DA5500	-	-	32.04	8.33	85.91	11.57	7.2

## Data Availability

The data are included in this manuscript and in its Supplementary Materials.

## Supplementary Materials

The following supporting information can be downloaded at: https://www.mdpi.com/article/10.3390/molecules28104021/s1, Figure S1: ^1^H NMR spectra of all intermediate products; Figure S2: FT-IR spectra of each product; Figure S3: The GPC spectra of DA copolymers; Figure S4: The relationship between apparent viscosity (η) and shear rate; Figures S5 and S6: The four groups of DA copolymer were cyclically scanned three times at different temperatures (80 and 120 °C) to obtain the curves of storage modulus (G′) and loss modulus (G″) with respect to angular frequency (a and d are DA2300, DA3200, DA4700, and DA5500). Table S1: The TGA data of each product; Table S2: Isothermal crystallization parameters of neat PPDO and DA copolymers; Table S3: The feeding and conditions of PPDO; Table S4: The feeding and conditions of PPDOER; Table S5: The feeding and conditions of PLA; Table S6: The feeding and conditions of PLAER; Table S7: The feeding and conditions of DA copolymer.

## Sample Availability

Samples of the compound are available from the authors.
