# Supplementary material for: A New Self-Healing Degradable Copolymer Based on Polylactide and Poly(p-dioxanone)
Source: Molecules. 2023 May 11;28(10):4021. doi: 10.3390/molecules28104021 (PMC10223814; doi:10.3390/molecules28104021)
Supplement: Supplementary file 1 [file molecules-28-04021-s001.zip › molecules-2361620-supplementary.pdf]

Supporting information for

A new self-healing degradable copolymer based on Polylactide and Poly(*p*-dioxanone)

Laifa Tong, Mi Zhou, Yulong Chen *, Kai Lu, Zhaohua Zhang, Yuesong Mu and Zejian He

Material Science and Engineering, College of Materials Science and Engineering, Zhejiang University of Technology, Hangzhou 310000, China

* Correspondence: chenyulong@zjut.edu.cn

Table S1. Homopolymer and copolymer data obtained from TG curves

Sample	$T_{5\%}^{a}$ (℃)	T_{max}^{b} (℃)	$T_{95\%}^{c}$ (℃)
PPDO3200	150	275	310
PLA3200	272	351	650
DA2300	238	305	468
DA3200	184	233, 334	427
DA4700	260	325	420
DA5500	215	307	360
PPDO/PLA	-	221, 336	-

[a] weight loss was 5%

[b] temperature corresponding to the maximum rate of weight loss

[c] weight loss was 95%

Table S2. Isothermal crystallization parameters of Neat-PPDO and D-A copolymers

Samples	n	k	$t_{1/2}$
Neat-PPDO	3.07	0.032	0.99 min
DA4700	2.86	0.644	2.85 min
DA5500	2.24	0.016	5.11min

Table S3. Conditions for the ring-opening polymerization reaction of PPDO

Samples	PDO/$SnOct_2$ (mol/mol)	PDO/ 1,4-Benzenedimethanol (mol/mol)	Temperature (℃)	Reaction time (h)	Yield (%)
PPDO2300	4000:1	50:1	80	24	78.3
PPDO3200	4000:1	70:1	80	24	81.5
PPDO4700	4000:1	100:1	80	24	74.9
PPDO5500	4000:1	150:1	80	24	82.5

Table S4. Conditions of the esterification reaction of PPDO

Samples	PPDO (g)	Furoic acid (g)	EDC (g)	DMAP (g)	Temperature (°C)	Reaction time (h)	Yield (%)
PPDO2300ER	1.5	0.1681	0.2875	0.0018	25	24	66.7
PPDO3200ER	2.0	0.1401	0.2396	0.0015	25	24	85.0
PPDO4700ER	2.0	0.0045	0.2698	0.0005	25	24	93.5
PPDO5500ER	1.9	0.071	0.1214	0.0007	25	24	83.5

Table S5. Conditions of the ring-opening polymerization of PLA

Samples	Lactide/$SnOct_2$ (mol/mol)	Lactide/1,4-Benzenedimethanol (mol/mol)	Temperature (°C)	Reaction time (h)	Yield (%)
PLA2300	4000:1	30:1	140	12	53.3
PLA3200	4000:1	40:1	140	12	52.1
PLA4700	4000:1	60:1	140	12	52.4
PLA5500	4000:1	70:1	140	12	55.4

Table S6. Conditions of the esterification reaction of PLA

Samples	PLA (g)	AMI (g)	EDC (g)	DMAP (g)	Temperature (°C)	Reaction time (h)	Yield(%)
PLA2300ER	1.5	0.2206	0.2500	0.0007	25	24	85.3
PLA3200ER	1.6	0.1933	0.2190	0.0006	25	24	80.1
PLA4700ER	2.8	0.1973	0.2236	0.0007	25	24	75.1
PLA5500ER	2.4	0.1353	0.1534	0.0005	25	24	78.6

Table S7. Conditions of the D-A reaction

Samples	PLAER (g)	PPDOER (g)	Temperature (°C)	Reaction time (h)	Yield (%)
DA2300	0.70	0.68	60	48	86.6
DA3200	1.00	1.04	60	48	87.5
DA4700	1.00	1.10	60	48	75.1
DA5500	1.23	1.01	60	48	70.3

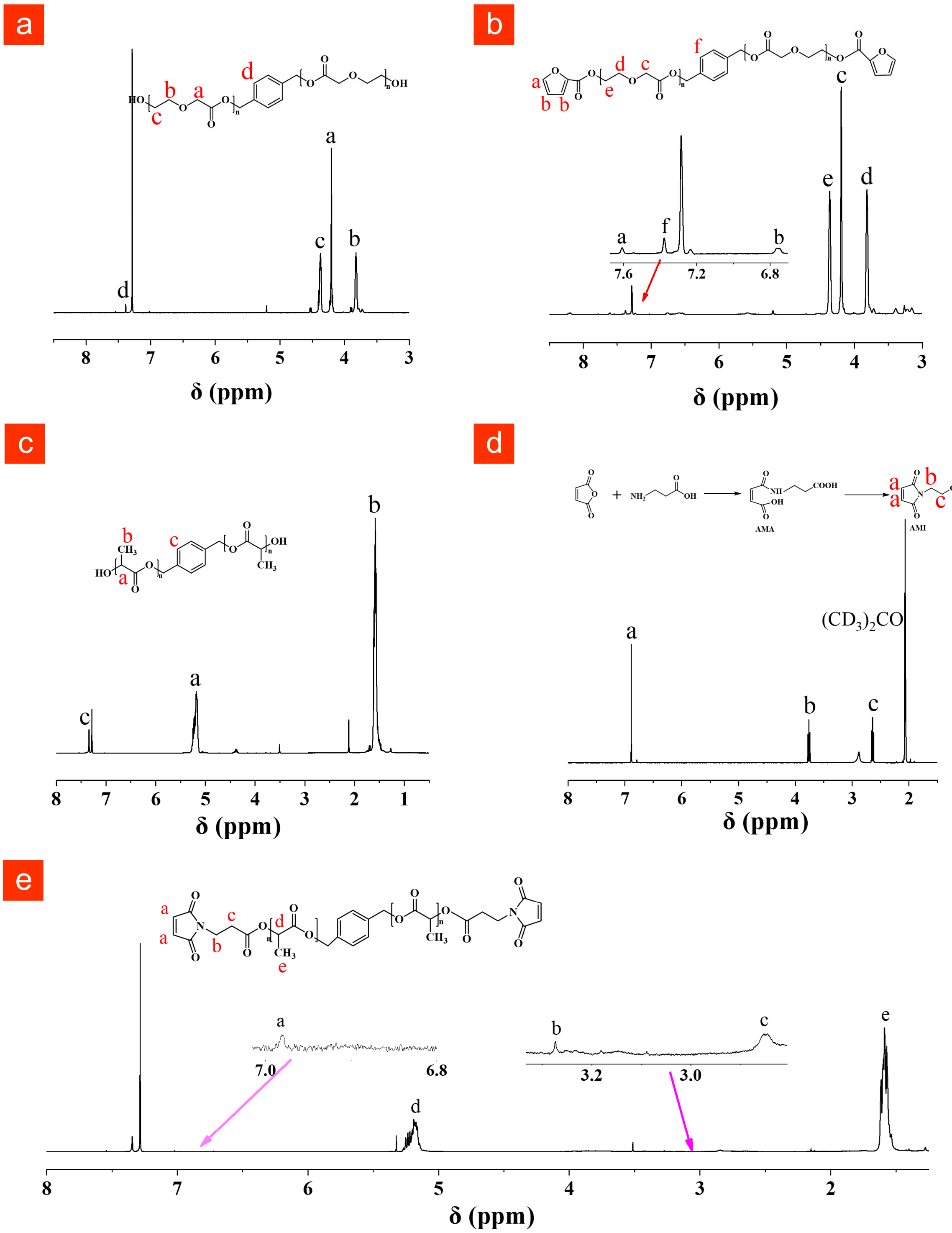

Figure S1. ^{1}H NMR spectra of the polymers: (a) PPDO; (b) PPDOER; (c) PLA; (d) AMI; (e) PLAER.

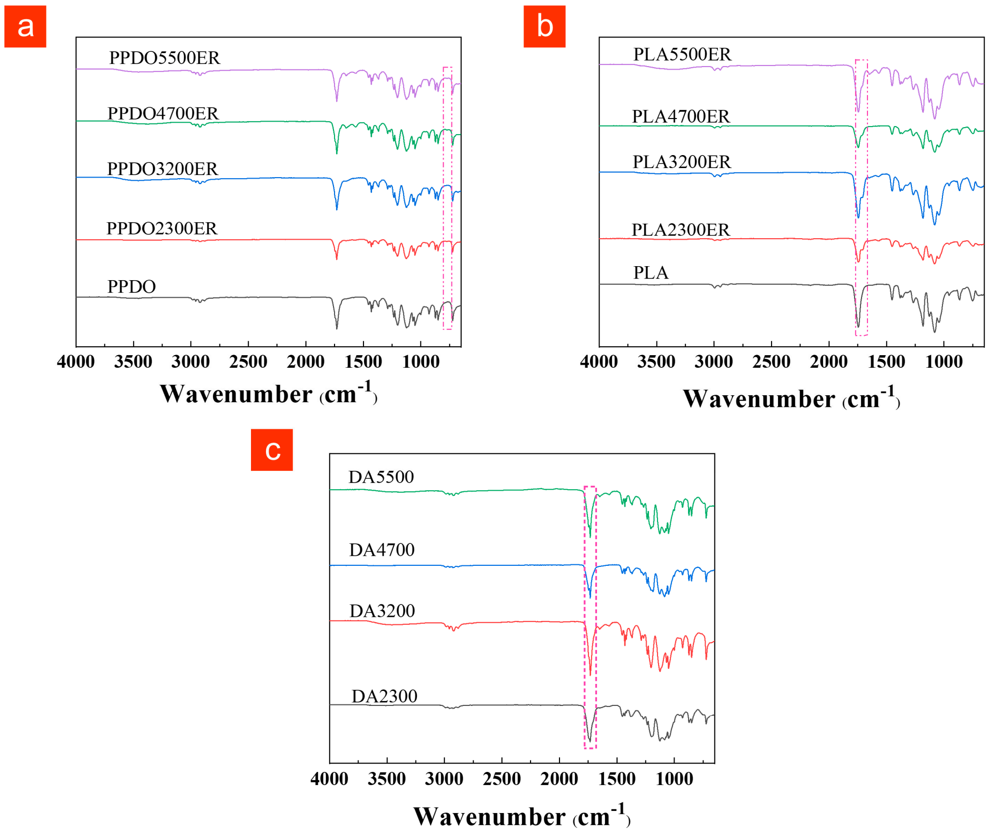

Figure S2. FT-IR spectrum of the polymers: (a) PPDO; (b) PLA; (c) D-A copolymer.

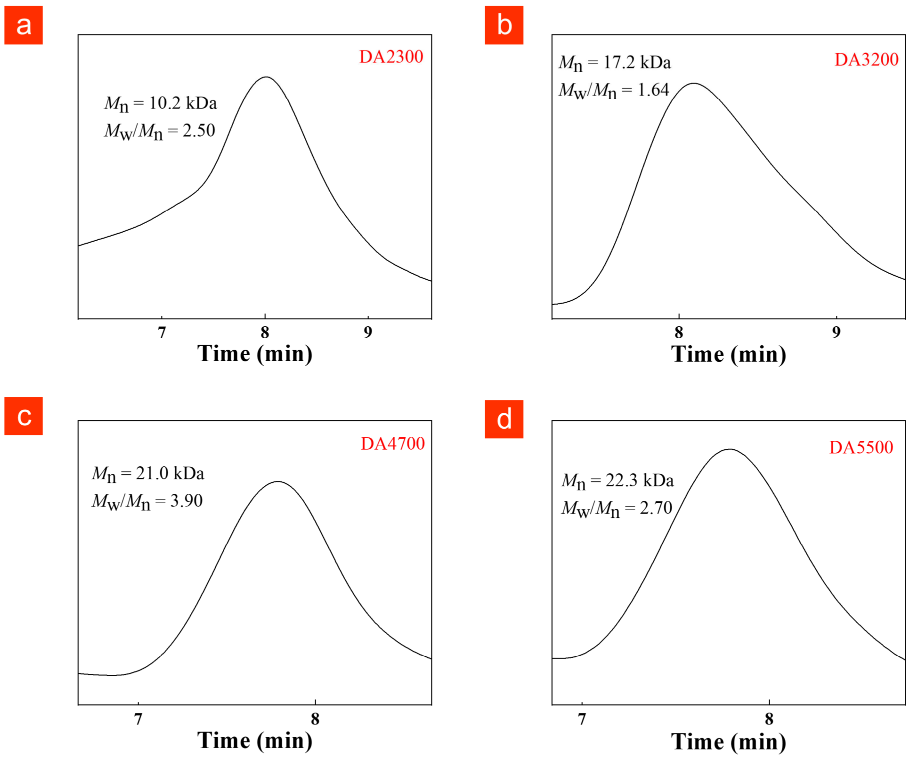

Figure S3. GPC spectrum of D-A copolymers: (a) DA2300; (b) DA3200; (c) DA4700; (d) DA5500.

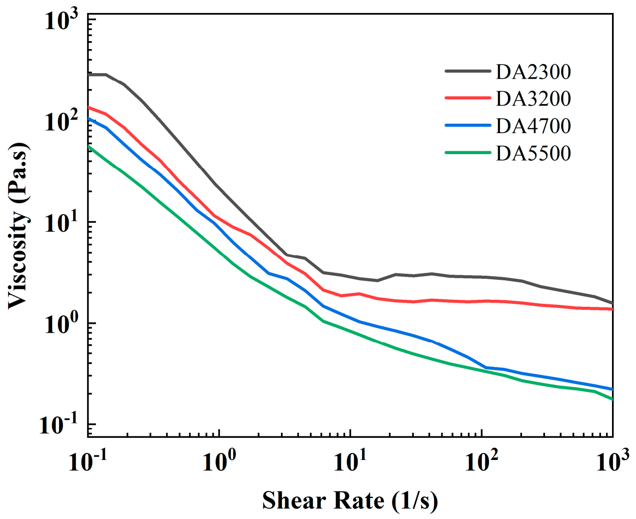

Figure S4. Curve of copolymer viscosity with shear rate

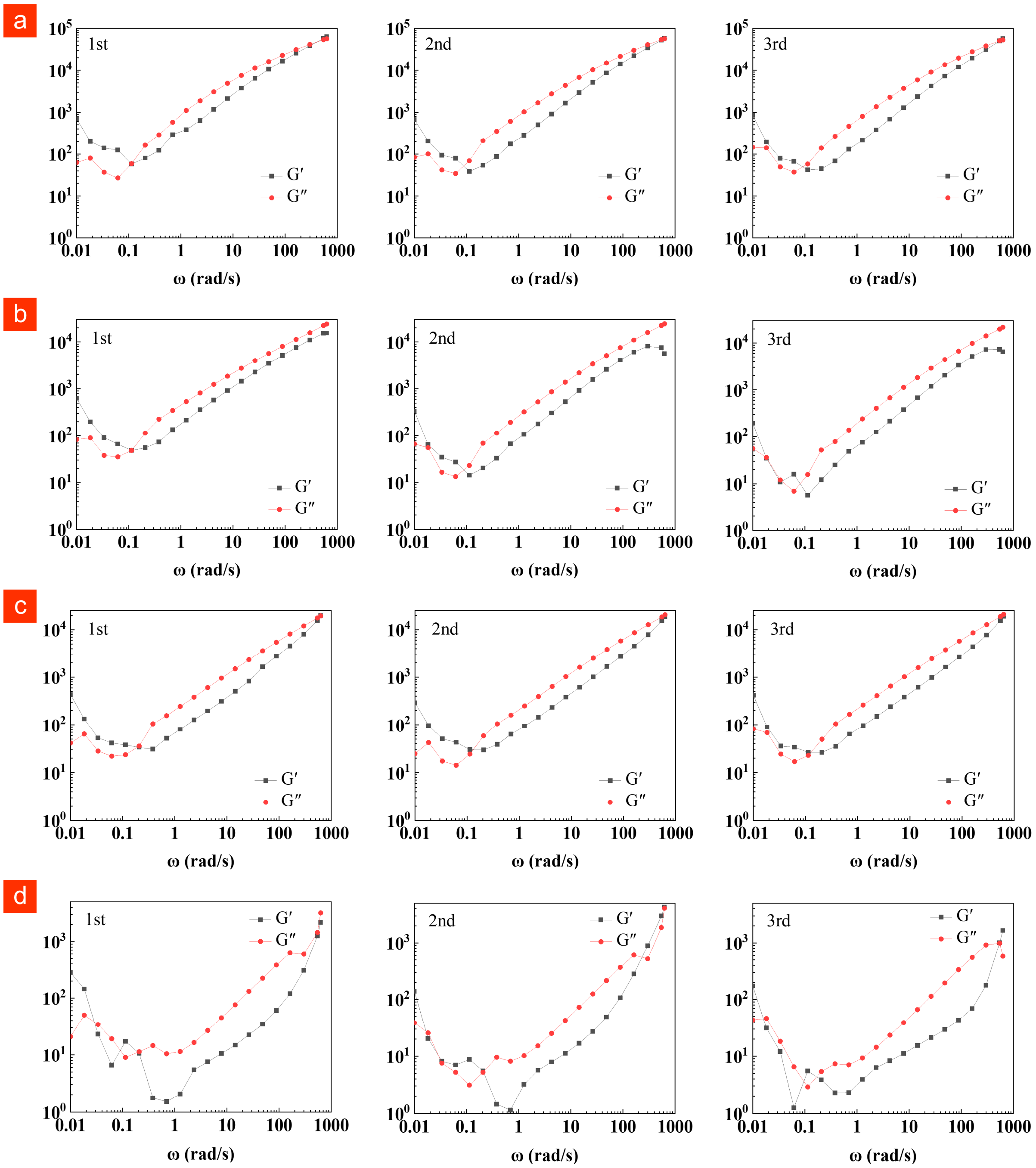

Figure S5. Angular frequency scanning curves of each product at 80 °C, (a) DA2300; (b) DA3200; (c) DA4700; (d) DA5500

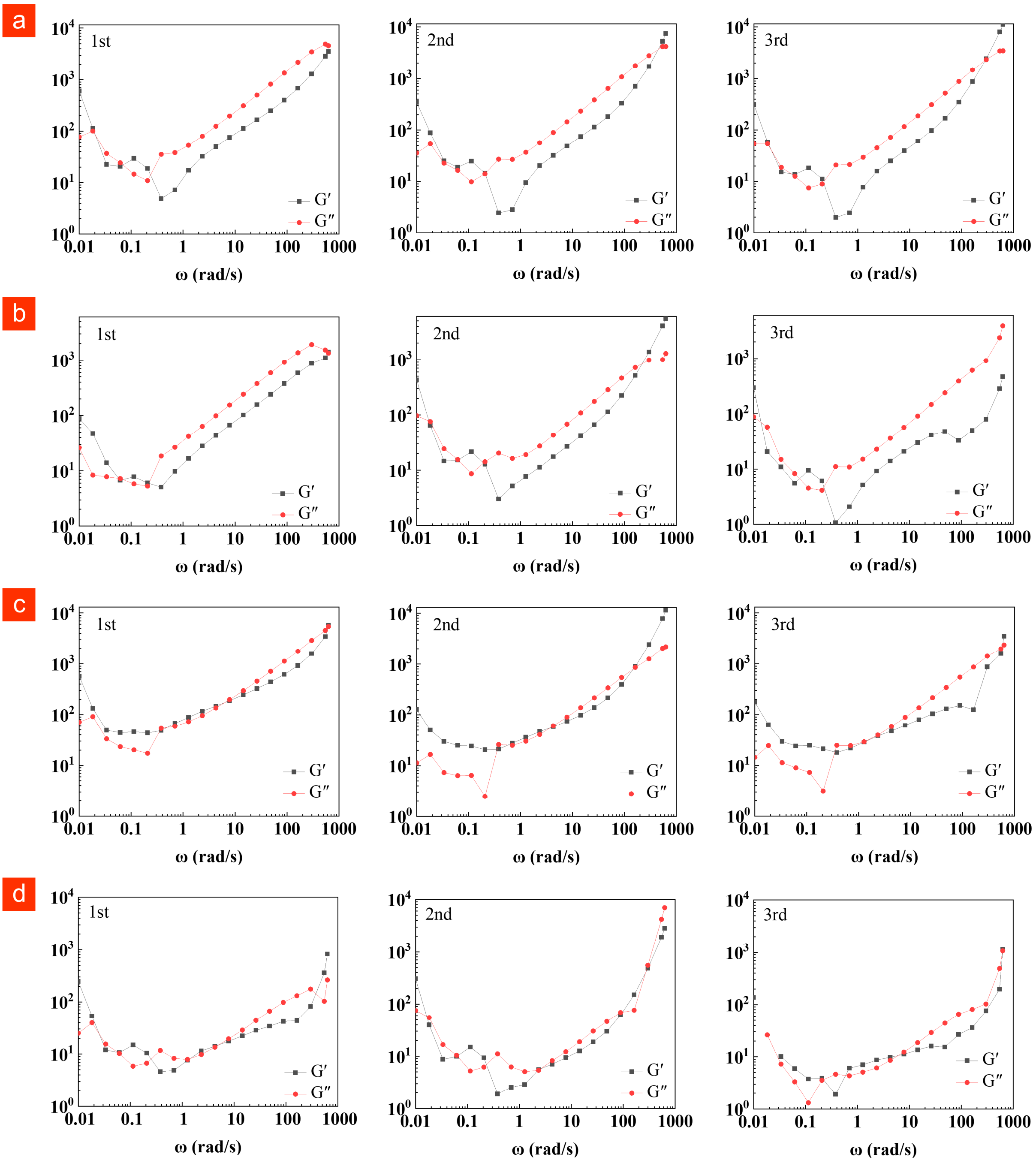

Figure S6. Angular frequency scanning curves of each product at 120 °C, (a) DA2300; (b) DA3200; (c) DA4700; (d) DA5500
